# Hyper-Crosslinked Carbohydrate Polymer for Repair of Critical-Sized Bone Defects

**DOI:** 10.1089/biores.2019.0021

**Published:** 2019-07-01

**Authors:** Plamena M. Koleva, James H. Keefer, Alexandria M. Ayala, Isabela Lorenzo, Christine E. Han, Kristen Pham, Stacy E. Ralston, Kee D. Kim, Charles C. Lee

**Affiliations:** ^1^ Molecular Matrix, Inc., West Sacramento, California.; ^2^ Department of Neurological Surgery, UC Davis School of Medicine, Sacramento, California.; ^3^ Department of Cell Biology and Human Anatomy, School of Medicine, University of California, Davis, California.

**Keywords:** bone graft substitute, critical-sized defect, hyper-crosslinked carbohydrate polymer, tissue engineering

## Abstract

This study evaluated the safety and efficacy of a novel hyper-crosslinked carbohydrate polymer (HCCP) for the repair of critical-sized bone defects in comparison to two alternative treatments: autologous bone and poly(lactide-co-glycolide) with hyaluronic acid (PLGA/HA). Bilateral critical-sized defects were created in the lateral femoral condyles of skeletally mature New Zealand White rabbits, and they were subsequently implanted with HCCP, PLGA/HA, or autologous bone in a randomized manner. Clinical and behavioral observations were made daily, and radiological and histopathological evaluations were performed at 4, 10, and 16 weeks postimplantation. Defects implanted with HCCP showed progressive bone regeneration and bridging of the defect without adverse histological events. No signs of infection or inflammation associated with the implant material were observed in all animals that received HCCP implantation. A radiographic assessment performed at 16 weeks post-implantation showed significantly higher bone density and volume in defects implanted with HCCP compared to PLGA/HA. No statistically significant difference was observed in bone density and volume between HCCP and autologous bone. These findings demonstrate that HCCP is biocompatible, osteoconductive, and capable of promoting bone regeneration *in vivo*; therefore, it is suitable for both tissue engineering and the repair of critical-sized bone defects.

## Introduction

Bone regeneration *in vivo*, as a treatment for bone defects such as those caused by fractures or osteotomies, remains a challenge for clinicians and patients, especially high-risk patients.^1^ Allograft and autologous bone are used in reconstructive surgeries to bridge bony gaps of the skeletal system and to promote bone regeneration in defects with compromised healing capacity. Historically, the standard treatment transitioned away from the use of allograft bone due to the risk of transmissible diseases, immune rejection by the host, and lot-to-lot variations.^2,3^ Autologous bone grafting became the gold standard due to the osteoinductive properties, reduced risk of disease transmission, and the inherent biocompatibility of a patient's own bone. More than 200,000 bone repair procedures are performed in the United States each year that utilize autologous bone harvested from the iliac crest^4^ as a source of graft material. Donor site morbidity, including infection, iliac wing fracture, loss of mobility, and chronic pain,^5,6^ as well as patient-specific complications, such as insufficient or nonviable donor bone, are among the most serious drawbacks of autologous bone harvest procedures.^7^ The significant limitations of allograft and autograft treatments have fueled the development of alternative materials to serve as a bone graft substitute with the intent to mitigate these issues.^5^

The ideal bone graft substitute must provide the benefits of autologous bone graft—biocompatibility, osteoconductivity, osteoinductivity, and osteogenicity—while eliminating the risks associated with harvesting autologous bone from patients. Desirable structural properties must include a physical scaffolding, resorbability, interconnected porous substructure, and pore sizes that are suitable for endogenous cellular infiltration and proliferation.^8^ The bone graft materials available on the market today do not wholly exhibit all properties of a desirable substitute to autograft.^8^ Biological substitutes, such as demineralized bone matrix, are certainly biocompatible, biodegradable, and osteoconductive; however, they lack osteoinductive and osteogenic properties.^7^ Synthetic substitutes, including metallics, ceramics, polymers, and calcium phosphates, have thus gained traction as effective alternatives. Synthetic biomaterials developed for use as bone graft substitutes are composed of substances such as hyaluronic acid (HA) and poly(lactide-co-glycolide) (PLGA).^8^

A novel hyper-crosslinked carbohydrate polymer (HCCP) shows clinical promise for the treatment of bone defects without the risk of immunogenic complications or adverse fibrotic tissue responses. Polysaccharides such as alginate and chitosan have been previously investigated for their role in bone repair and regeneration for a couple of decades.^9,10^ However, there have not yet been any polysaccharide-based technologies developed and utilized for bone regeneration in a clinical setting. HCCP has been used for *in vitro* applications for generating organoids derived from stem, progenitor, and cancer cells,^11,12^ but it has yet to be explored for a bone repair application. In this study, for the purpose of bone repair/regeneration application, we investigated the safety and efficacy of HCCP for the repair and regeneration of bone in a critical-sized bone defect model.

## Materials and Methods

### Animal model

The New Zealand White (NZW) rabbit model for critical-sized bone defects has been previously established.^13^ Male NZW rabbits (*n* = 27; 3.5–4.5 kg; >6 months old) were included in the study after confirmation of skeletal maturity via x-ray imaging of closed physes of the distal femur and proximal tibia. Rabbits were housed individually in stainless steel cages with controlled environmental conditions. Commercial pelleted laboratory feed (LabDiet, St. Louis, MO) was provided daily, and fresh timothy hay and purified tap water were provided *ad libitum*. All animal procedures were performed in accordance with the Animal Welfare Act and were approved by the Institutional Animal Care and Use Committee (IACUC).

### Surgical procedure

After an acclimation period of at least 7 days, rabbits were randomized to receive bilateral implantation of HCCP, autograft, or PLGA/HA. A pre-anesthetic mixture of ketamine hydrochloride (20 mg/kg) and midazolam (2 mg/kg) was administered subcutaneously to elicit mild sedation. The left and right lateral hindlimbs of each rabbit were clipped. In groups receiving autograft treatment, the dorsum was shaved at the iliac crest site. Surgical plane anesthesia was induced and maintained with isoflurane (1–5%) volatilized with pure oxygen (2 L/min) administered via a nasal gas mask. The surgical sites were prepared with 10% povidone-iodine and 70% ethanol alternating scrub and were sterile draped with the animal in the lateral decubitus position.

The lateral femoral condyle was palpated and accessed by a longitudinal incision. The superficial and deep fascia were identified and incised separately; then, a 10.0-mm segment of bone was exposed by dissection between the overlaying musculature. The periosteum was incised and stripped from the bone by scraping with a periosteal elevator. Using a high-speed drill with 2.0-mm burr (Medtronic, Minneapolis, MN), a 7.0-mm diameter by 10.0-mm depth cylindrical critical-sized bone defect was created in a uniform and reproducible manner in the center of the lateral femoral condyle. The bone defect was filled with ∼1.0 cc of HCCP, autologous bone, or PLGA/HA and sealed with bone wax to prevent implant migration and soft tissue infiltration. In groups receiving autograft, ∼1.0 cc corticocancellous autologous bone was harvested from the iliac crest immediately before implantation. Autologous bone was morcelized into 1.0–4.0-mm pieces by using a rongeur and reserved for implantation in the same animal. The superficial and deep fascia were closed by using 4–0 absorbable PDS-II suture (Ethicon, Cincinnati, OH), and the skin was closed by using surgical staples. Animals were rotated to the contralateral side where the bone defect and implantation procedures were repeated with the assigned treatment material.

Routine clinical observations were conducted daily postoperatively. No fixation devices were used, and animals were allowed to move freely in individual cages. The animals were provided with Buprenex (buprenorphine, 0.01–0.05 mg/kg, subcutaneous) for pain management, Carprieve (carprofen, 5 mg/kg, subcutaneous) for anti-inflammation, and Baytril (enrofloxacin, 5–15 mg/kg, subcutaneous) for postoperative antibiotic prophylaxis. After recovery, animals were monitored daily for incision healing, activity level, mobility of the hindlimbs, and gait. Range of movement in the hindlimbs and any evidence of macroscopic reaction of the subcutaneous tissue to the implant materials were noted before euthanasia. Animals were euthanized by using Euthasol (pentobarbital sodium and phenytoin sodium, 100 mg/kg, intravenous) at 4, 10, and 16-week time points.

### High-resolution micro-computed tomography assessment of fusion

Right and left femurs were harvested, digitally photographed, and placed in 10% phosphate-buffered formalin for at least 72 h before transfer to 70% ethanol. Micro-computed tomography (μCT) imaging was performed (Veterinary Orthopedic Research Lab, University of California, Davis, CA) on femur samples by using a 20-mm-long region of interest contoured to fit the bone originating at the distal end imaged (70 kVp, 114 μA, 300 ms integration time, average of three images) by using a high-resolution μCT specimen scanner (μCT 35, Scanco Medical, Bassersdorf, Switzerland) with a 0.5-mm aluminum filter and 18.5-μm voxel resolution. Serial tomograms were reconstructed from raw data of 500 projections per 180° by using a cone beam filtered back projection algorithm adapted from Feldkamp et al.^14^

### Histological processing

After μCT evaluation, femurs were sent to the School of Medicine Histology Core at Washington University (St. Louis, MO) for histological processing. Femurs were cut to remove the excess shaft; then, the condyle heads were bisected longitudinally and embedded in methyl methacrylate. The blocks were thin sectioned from the midline of the defect outward and stained with Toluidine Blue. Sections of each implant were examined microscopically (Olympus BX61 Light Microscope) for inflammatory response and for measurement of peri-implant fibrosis. The extent of inflammatory response was quantified by assessing the presence of inflammatory cells (polymorphonuclear cells, lymphocytes, macrophages, and foreign body giant cells), fibrin, exudate, necrosis, and vascularization. Sections were also evaluated for new bone growth and integration with the implant material at each time point. Draining local lymph nodes (inguinal and popliteal) were collected for paraffin embedding and staining with hematoxylin and eosin; then, they were examined microscopically for atypical reactive response (lymphocyte or macrophage infiltration).

### Statistical analysis

Results were reported as the mean ± standard error of the mean and calculated by using Microsoft Excel (Microsoft, Redmond, WA). Statistical significance (*p* < 0.05) was determined by analysis of variance or two-sided Student's *t*-test analysis.

## Results

### Clinical observations

**FIG. 1.**
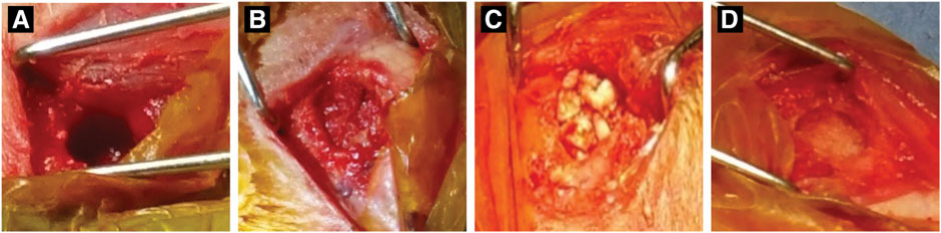
Critical-sized bone defect in femoral condyle. A defect sized 7 mm diameter by 10 mm depth was created in the femoral condyle of rabbits bilaterally **(A)**. The defect was filled with autologous bone **(B)**, PLGA/HA **(C)**, or HCCP **(D)**. HCCP, hyper-crosslinked carbohydrate polymer; PLGA/HA, poly(lactide-co-glycolide)/hyaluronic acid.

All animals that received autologous bone (Fig. 1B), PLGA/HA (Fig. 1C), or HCCP (Fig. 1D) underwent the surgical procedures without any adverse clinical episodes. Complete soaking of HCCP with blood/bone marrow was observed immediately after implantation compared with PLGA/HA, which showed incomplete uptake of blood/bone marrow (Fig. 1). Overall, animals implanted with HCCP showed subcutaneous abscess (*n* = 1) and non-weight bearing behavior (*n* = 2) during the entire course of the study compared with PLGA/HA (subcutaneous abscess [*n* = 1], non-weight bearing [*n* = 3]) and autologous bone (non-weight bearing [*n* = 2]). The incidence of adverse events in animals in the HCCP group was not higher than the other groups. After filling the defect with the graft materials (autologous bone, PLGA/HA, HCCP), the surgical site was covered with bone wax, which provided an impermeable barrier between the graft and overlaying tissues. Bone wax was still present at study term, and the graft materials did not come into contact with overlaying tissues. Thus, we conclude that the tissue response observed adjacent to the implant sites may show complications that are inherent to the surgical procedure performed on these animals.

### Radiographical evaluation of implant sites

**FIG. 2.**
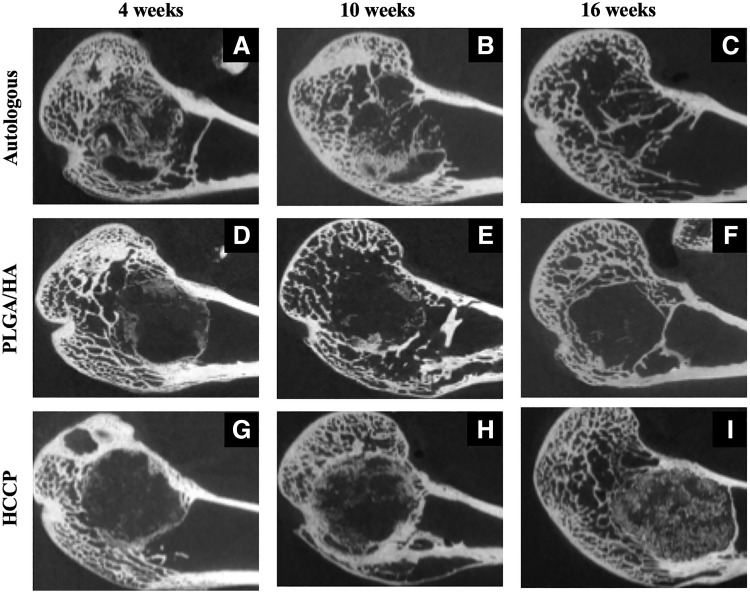
μCT imaging of implant sites. Femurs from animals in each group were imaged by μCT at 4 **(A, D, G)**, 10 **(B, E, H)**, and 16 **(C, F, I)** weeks postimplantation of autologous bone **(A–C)**, PLGA/HA **(D–F)**, or HCCP **(G–I)**. μCT, micro-computed tomography.

High-resolution μCT was performed on femurs harvested from all animals at 4, 10, and 16 weeks postimplantation. At 4 weeks postimplantation, defect sites implanted with autologous bone showed a significant amount of bone in the defect (Fig. 2A). The defect sites filled with PLGA/HA (Fig. 2D) and HCCP (Fig. 2G) showed evidence of calcification in the periphery at 4 weeks without signs of bridging. At 10 and 16 weeks postimplantation, a slight decline in the overall amount of bone was observed in defect sites implanted with autologous bone (Fig. 2A–C). No further bone growth was observed beyond 4 weeks in defect sites implanted with PLGA/HA (Fig. 2D–F). Defect sites implanted with HCCP continued to show bridging of the critical-sized defect through 10 and 16 weeks postimplantation (Fig. 2G–I).

**FIG. 3.**
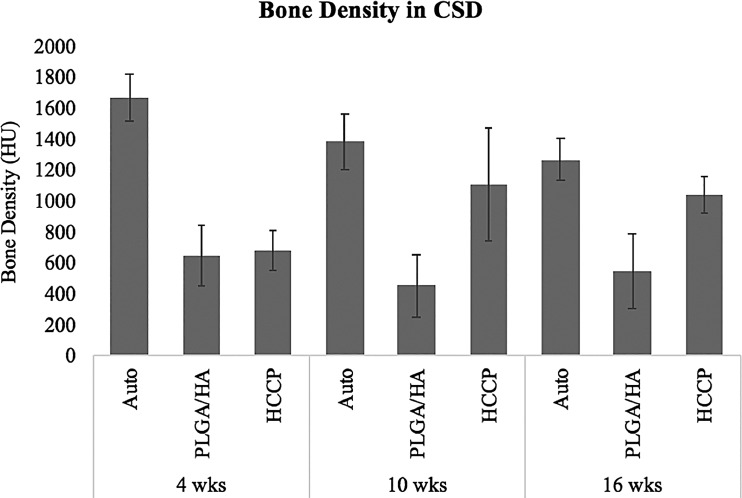
Bone density in critical-sized defect. ROI was defined with each critical-sized defect, and HU was measured. HU values were compared between animals assigned to different study groups. Auto, autologous bone; HU, hounsfield units; ROI, region of interest.

**FIG. 4.**
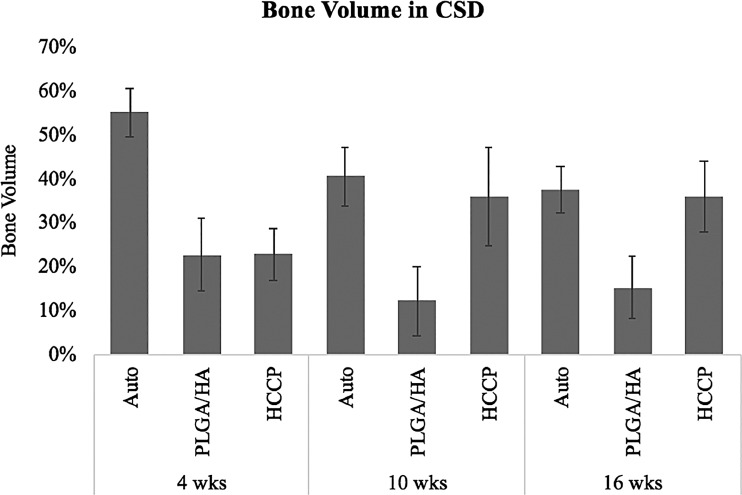
Bone volume in critical-sized defect. Percent bone in the total volume of each critical-sized defect was compared between animals assigned to different study groups. Auto, autologous bone.

Hounsfield units (HU) were quantified in the critical-sized defects at all time points in all animals (Fig. 3). Defect sites implanted with autologous bone showed 1669.6 ± 152.4 HU at 4 weeks and declined to 1270.4 ± 135.8 HU at 16 weeks postimplantation (*p* < 0.05). At 4 weeks postimplantation, defects implanted with autologous bone showed a significantly higher HU compared with PLGA/HA (652.5 ± 194.8 HU; *p* < 0.05) or HCCP (682.0 ± 130.4 HU; *p* < 0.05). No significant difference in HU was observed in defect sites implanted with PLGA/HA between 4 weeks (652.5 ± 194.8 HU) and 16 weeks (548.5 ± 236.8 HU; *p* > 0.05). However, a significant increase in HU was observed in defect sites implanted with HCCP between 4 weeks (682.0 ± 130.4 HU) and 16 weeks (1039.9 ± 119.1 HU; *p* < 0.05). At 16 weeks, the defect sites implanted with autologous bone (1270.4 ± 135.8 HU) and HCCP (1039.9 ± 119.1 HU) showed a significantly higher HU than PLGA/HA (548.5 ± 236.8; *p* < 0.05). No significant differences were observed between autologous bone and HCCP groups at 16 weeks (*p* > 0.05). Similar outcomes were observed when the total bone volumes were compared between the groups (Fig. 4).

### Histological evaluation of implant sites

**FIG. 5.**
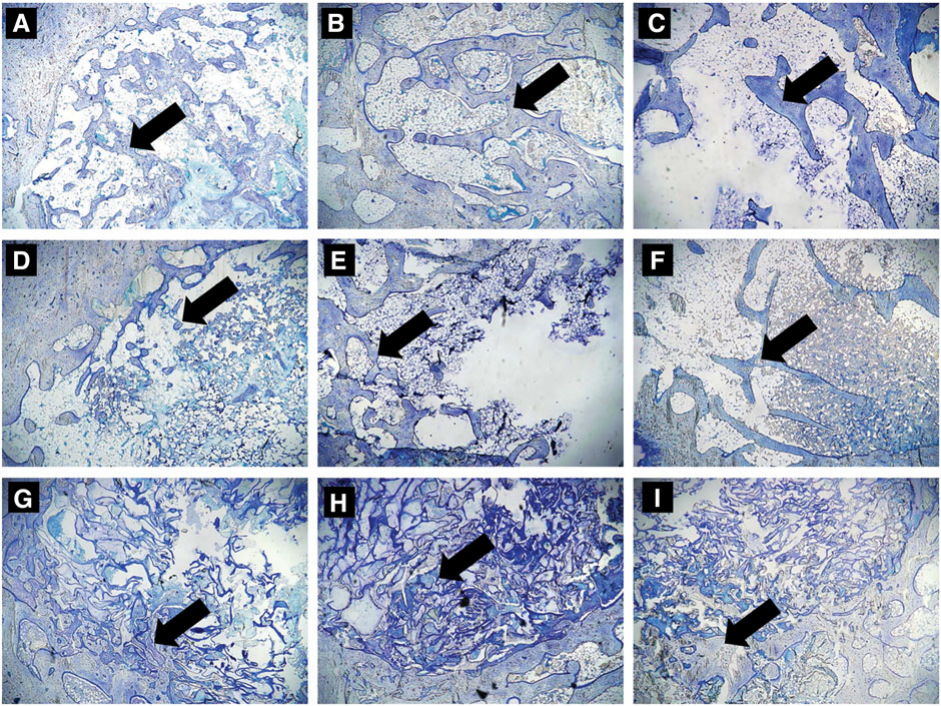
Histological evaluation of implant sites. Sections of femur samples embedded in methyl methacrylate were stained with Toluidine Blue for histological evaluation at 4 **(A, D, G)**, 10 **(B, E, H)**, and 16 **(C, F, I)** weeks postimplantation of autologous bone **(A–C)**, PLGA/HA **(D–F)**, and HCCP **(G–I)**. Arrow indicates bone in critical-sized defect. Magnification = 10 × .

Numerous implant trabecular bones were observed in the critical-sized femoral condyle defects of animals implanted with autologous bone (Fig. 5A) at 4 weeks postimplantation. Animals implanted with PLGA/HA (Fig. 5D) or HCCP (Fig. 5G) showed minimal bone formation in the periphery of the defect sites. As observed with μCT, a visible, progressive decline in the amount of bone in the defect was noted in animals implanted with autologous bone at 10 (Fig. 5B) and 16 weeks (Fig. 5C) postimplantation. Animals implanted with PLGA/HA showed growth of new bone restricted to the peripheral region of the defect and a minimal amount of the implant material at 10 (Fig. 5E) and 16 (Fig. 5F) weeks. Growth of trabecular bone was observed in the defects implanted with HCCP at 16 weeks postimplantation (Fig. 5I).

**FIG. 6.**
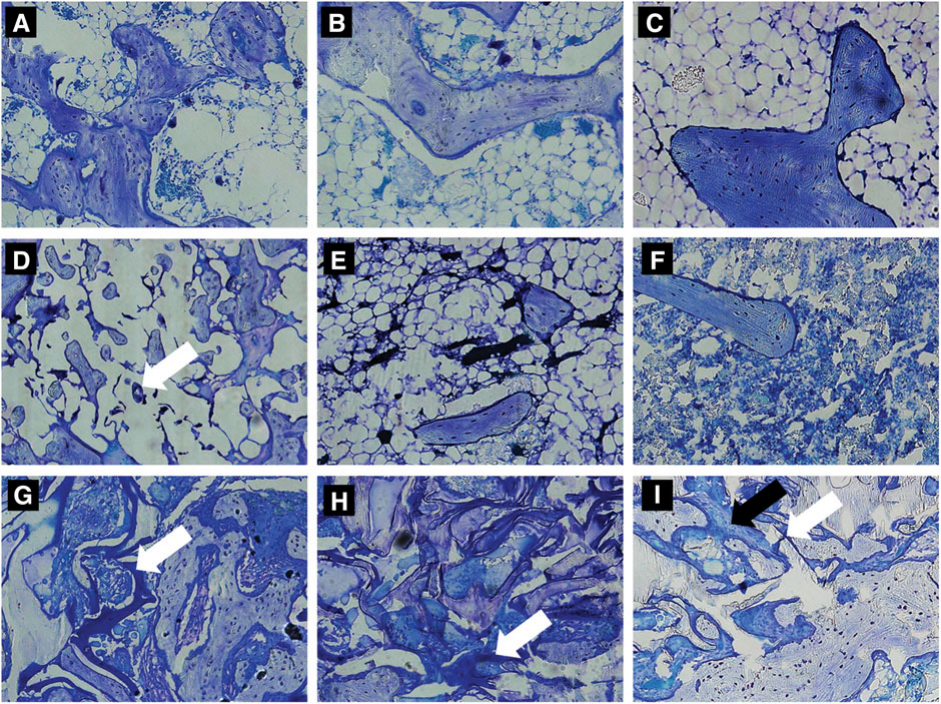
Histological evaluation of implant materials. Sections of femur samples were evaluated for residual implant material and adverse histological findings at 4 **(A, D, G)**, 10 **(B, E, H)**, and 16 **(C, F, I)** weeks postimplantation of autologous bone **(A–C)**, PLGA/HA **(D–F)**, and HCCP **(G–I)**. White arrow indicates implant material; black arrow indicates osteoblasts. Magnification = 20 × .

Higher magnification images showed an absence of severe adverse histological events in all study groups (Fig. 6). Minimal fragmented, residual PLGA/HA implant material was observed (Fig. 6D, white arrow). No PLGA/HA implant material was observed beyond 4 weeks postimplantation. However, the HCCP implant material was still visible and intact at 4 weeks postimplantation (Fig. 6G, white arrow). Noticeable degradation of the HCCP implant material was observed over the course of the study (Fig. 6G–I; white arrow). Osteoblasts were observed around new bone at the HCCP implant (Fig. 6G, black arrow). Three cases of mild lymphocytic infiltration and one case of mild fibrosis were observed at the implant site in animals implanted with autologous bone. Animals implanted with PLGA/HA showed 10 cases of mild lymphocytic infiltration and 2 cases of fibrosis. No adverse histological events were observed with HCCP.

## Discussion

Normal bone is remarkably regenerative and maintains homeostasis through various cellular components (osteoblasts, osteoclasts), growth factors, extracellular matrices, and vasculature.^15^ These factors must work together in a well-orchestrated manner to initiate and maintain the complex physiological process of repairing damaged or missing bone. An autologous bone graft contains osteogenic cells, osteoinductive factors, and osteoconductive matrices, which provide an ideal solution to issues that occur in the skeletal system. However, due to the morbidity associated with harvesting autologous bone, the use of bone graft substitutes has been increasing over the past several decades.

Parikh described the past, present, and future of bone graft substitutes in 2002 and categorized the then-current substitutes broadly into calcium phosphate/sulfate, ceramics, tricalcium phosphate, collagen, non-biologic substrates (e.g., polylactic and polyglycolic acid polymers), demineralized bone matrix, bone morphogenetic proteins, and other growth factors.^16^ Parikh also proposed stem cells (tissue engineering) and gene therapy as the future direction. However, no stem cell or gene therapy approach has been approved by the U.S. FDA for bone applications, and all of the bone graft substitutes listed earlier are still used with minimal improvements over the past 17 years since the publication by Parikh.^16^ This study highlights the utilization of a new bone graft substitute, HCCP, that is unique in its chemical composition, radiolucency, high biocompatibility, and resorption-to-regeneration rate profile compared with other substitutes currently available for treating patients with bone-related health issues.

Several studies have reported the utility of carbohydrate or saccharide-based polymers/scaffolds for bone regeneration.^17–20^ Carbohydrate-based polymers/scaffolds are believed to participate in bone regeneration via their structural similarities to glycosaminoglycans (GAGs, long unbranched polysaccharides); complete resorption through degradation by lysozymes *in vivo*; lack of chronic inflammatory response at the implant site^16^; and high charge density under mild physiological conditions for interaction with proteins/factors, cells, and tissues. Specifically, GAGs have been shown to enhance osteoblastic differentiation of bone marrow-derived mesenchymal stem cells with upregulation of genes involved in osteogenesis.^21^ Genetic enrichment of GAGs has led to an increase in bone mass and inhibition of resorption, modulating the crosstalk between osteoblasts and their microenvironment via the Wnt-β-catenin–T cell factor signaling pathway.^22^ Thus, carbohydrate-based polymers may mimic the function of GAGs in bone repair and regeneration *in vivo*. Consistently, the results of our study indicate that HCCP promotes a significantly higher level of bone regeneration compared with synthetic PLGA/HA in a critical-sized bone defect in the rabbit model. HA, which is a component of PLGA/HA, may have played a role in early bone regeneration postimplantation as observed in this study. However, most of PLGA degrades with a few polymeric spots remaining at 4 weeks postimplantation (∼50% at 4 weeks, no polymer found at 10 weeks), indicating a significant degradation in the first 1 month after surgery.^23^ Considering much longer time needed for natural bone regeneration and healing (3–6 months), HCCP with a longer degradation profile (3–6 months) may provide a better osteoconductive environment than other substitutes with shorter or longer degradation profiles.

Carbohydrates are naturally found in the body and do not elicit the immune response. HCCP degrades most likely to mono- and/or oligomeric saccharides via enzymatic hydrolysis, and the level of degraded products released from the implant site may be negligible, considering the time course of HCCP degradation *in vivo* (∼6 months). Thus, it is unlikely for HCCP to trigger the immune system or cause systemic toxicity postimplantation in patients. These properties provide a significant advantage over synthetic resorbable polymers such as poly(glycolic acid) or poly(lactic acid) that can cause severe inflammation by the release of acidic degradation by-products.^24^ Similarly, in addition to acidic degradation, PLGA has been shown to elicit an inflammatory response after uptake by macrophages, resulting in an increase in proinflammatory factors such as TNF-α^25^ and fibrotic encapsulation after implantation.^26^ The immunogenicity of other biologics such as allogeneic bone graft substitutes and xenogenic collagen has been well established.^5^

Pseudoarthrosis is one of the most clinically significant condition after implantation of a bone graft substitute that may require revision surgery. An objective correlation between the degree of pseudoarthrosis and persistent or recurrent pain may be important in determining the need for revision surgery. CT provides a powerful tool for evaluating bone fusion and pseudoarthrosis, showing a significantly higher specificity for detecting nonunion compared with plain radiography. However, prior studies have estimated the accuracy of diagnosing pseudoarthrosis by CT at ∼60–80% of the findings by surgical exploration.^27^ The discrepancy between CT and surgical exploration may be attributed to artifacts from adjacent instrumentations. More importantly, ceramic-based or mineralized bone graft substitutes composed of substances such as calcium sulfate, hydroxyapatite, or tricalcium phosphate are intrinsically radiopaque, typically appear denser than adjacent patient bone, and interfere with the evaluation of ingrowth of new bone into the graft material or the degree of nonunion. Thus, it is important to note that a radiolucent bone graft substitute such as HCCP and PLGA/HA provides a significant clinical benefit of allowing evaluation of bone ingrowth by CT as observed in this study.

## Conclusions

Osteoconductive matrices or scaffolds, osteoinductive signals, and osteogenic cells are required for healthy bone healing. Efforts have been made to introduce all of these factors to promote efficient bone regeneration *in vivo*.^28–31^ However, it may also be possible to initiate and maintain osteogenesis during the course of bridging a critical-sized defect in patient bone^32^ using a novel polymeric material such as HCCP that can mimic the native bone microenvironment. HCCP supports the proliferation and differentiation of bone stem/progenitor cells without the need for additional osteoinductive or osteogenic factors. As a bone graft substitute, HCCP provides a new opportunity to deliver a safe and effective alternative to autologous bone graft, or the combination approach, while maintaining the ability to monitor bone regeneration radiographically and without eliciting the host immune response.

## Abbreviations Used

μCT: micro-computed tomography
Auto: autologous bone
GAG: glycosaminoglycan
HCCP: hyper-crosslinked carbohydrate polymer
HU: hounsfield units
IACUC: Institutional Animal Care and Use Committee
NZW: New Zealand White
PLGA/HA: poly(lactide-co-glycolide)/hyaluronic acid
ROI: region of interest
